# Supplementary material for: Generalized Wardowski type contractive mappings in b-metric spaces and some fixed point results with applications in optimization problem and modeling biological ecosystem
Source: PLoS One. 2024 Dec 18;19(12):e0313033. doi: 10.1371/journal.pone.0313033 (PMC11654975; doi:10.1371/journal.pone.0313033)
Supplement: Attachment — Submitted filename: Review comments on PONE-D-24-31370.pdf [file pone.0313033.s002.pdf]

Generalized Wardowski Type Contractive Mappings in b-Metric Spaces and Some Fixed Point Results with Applications in Optimization Problem and Modeling Biological Ecosystem

Authors: Maryam Iqbal , Afshan Batool , Aftab Hussain , Hamed Al-Sulami

The paper claims to introduces a generalized Wardowski-type quasi-contraction, termed $\beta - (\theta, \vartheta)$, within the context of b-metric spaces. The work establishes fixed point results using this new contraction and confirms its validity by identifying it as a generalization of Wardowski's quasi-contraction. The study further claims to illustrate the application of these results to biological ecosystem modeling, based on Nadler's work, and to an optimization problem. While the mathematical aspects of the paper may have some merit, the claimed applications are speculative rather than genuine. To present real applications, the authors should develop concrete models and revise these sections with a more constructive application(s) or remove the application parts altogether. The paper is also poorly organized and written in weak English. Therefore, I recommend major revision, and here are my further observations:

i. In the introduction, the authors used the phrase `which is referred as' this should be `which is referred to as', please correct this throughout the manuscript.
ii. Add more related literature in the introduction and discuss in depth about each work.
iii. I was expecting to see the organization or structure of the paper at the end of the introductory section but there is none. Please, include this.
iv. The first sentence in the Preliminaries section is grammatically not correct, please correct it.
v. Definition 2.1 is also poorly written.
vi. In the entire paper the name 'Nadler' was written wrongly almost everywhere, see, for example second line under main results and the subsection 3.1.
vii. Optimization was also written as optimisation in some parts of the paper, please be specific and rewrite them correctly, see, for example the title of the paper and subsection 3.2.
viii. I could not find how in subsection 3.1, a biological ecosystem was modeled using Nadler's work, please put a genuine application or remove this claim.
ix. Subsection 3.2 is not an application rather than just speculations, please remove it.
x. The paper should be thoroughly reorganized, proofread, and spell-checked.
