# Supplementary material for: Generalized Wardowski type contractive mappings in b-metric spaces and some fixed point results with applications in optimization problem and modeling biological ecosystem
Source: PLoS One. 2024 Dec 18;19(12):e0313033. doi: 10.1371/journal.pone.0313033 (PMC11654975; doi:10.1371/journal.pone.0313033)
Supplement: Attachment — Submitted filename: Response Letter Reviewer 1 Ploss One.pdf [file pone.0313033.s003.pdf]

Generalized Wardowski Type Contractive Mappings in b-Metric Spaces and Some Fixed Point Results with Applications in Optimization Problem and Modeling Biological Ecosystem

First of all, the authors would like to thank dear reviewer for the constructive and useful comments to improve the quality of the paper. We tried to modify the paper based on the comments and we hope that this version will be acceptable for dear reviewer and respected Editor.

Reviewer#1

The paper is written badly as it is very difficult to read due to non-standard symbols. There is no novelty in the paper as more generalized spaces are available in the literature. Authors has claimed for application but there is no details studies have been done. The paper has NO merit to publish in any SCI/SCOPUS journals.
Paper is rejected from bottom.

Answer: Thank you for your detailed feedback on our manuscript. We appreciate your insights and the time you have taken to review our work. We have carefully considered your comments and have made substantial revisions to address the concerns raised. Below, we outline the specific actions we have taken in response to each of your points.

We understand the concerns about the clarity of the symbols used in the paper. We have revised the manuscript to use standard mathematical notation and symbols. We believe these changes significantly enhance the readability and accessibility of the paper. The revised manuscript has been carefully checked to ensure that all symbols and notations are standard and consistent.

We have expanded our discussion to highlight the specific contributions and novelty of our work in the context of existing literature. We emphasize that while similar spaces have been studied, our approach provides new insights and practical

applications not covered by previous work. We have included a more detailed comparison with related studies to clarify the unique aspects of our contribution.

We acknowledge the need for more detailed application studies. To address this, we have included additional sections in the revised manuscript that elaborate on the practical applications of our findings. These sections provide concrete examples and case studies that demonstrate the relevance and utility of our work. We believe these additions strengthen the overall contribution of the paper.

We have made significant revisions to address the concerns about the paper's merit. By incorporating the above improvements, we believe the manuscript now meets the standards expected for publication in SCI/SCOPUS journals. We are confident that the revised version of the paper makes a meaningful contribution to the field and provides valuable insights.

We hope that these revisions address your concerns and that the manuscript now meets the necessary criteria for publication. We appreciate your feedback and look forward to your further review of the revised paper.

Thank you once again for your time and consideration.
