# Supplementary material for: Generalized Wardowski type contractive mappings in b-metric spaces and some fixed point results with applications in optimization problem and modeling biological ecosystem
Source: PLoS One. 2024 Dec 18;19(12):e0313033. doi: 10.1371/journal.pone.0313033 (PMC11654975; doi:10.1371/journal.pone.0313033)
Supplement: Attachment — Submitted filename: PLOS ONE Report.docx [file pone.0313033.s001.docx]

**Generalized Wardowski Type Contractive Mappings in b-Metric Spaces and Some Fixed Point Results with Applications in Optimization Problem and Modeling BiologicalEcosystem**

**Key Contributions**

1. The paper proposes a β-(θ, ϑ) a generalizedWardowski type quasi-contractionto split fixed point problems.
2. First, authors use our new contraction to provide typical fixed point results, and then we show that there is a generalized quasi-contraction of the Wardowski type, confirming the validity of our results.
3. Second they demonstrate the modelling of biological ecosystems using Nadler’s work and the application of our results to an optimization problem.
4. In order to demonstrate the effectiveness of results, and present a comparison between result and Nadler’s work.
5. Theorem has important applications in computer science, biology, and economics, among other domains. Biological ecosystem modelling.

**Areas of Application**

- Computer vision
- Computer graphics
- Image restoration
- Complex 3D Models

**Comments and suggestions:**

**Abstract:**

Generalised should be generalized, optimisation should be optimization,

Introduction: “Banach withdrawal standard” revise this sentence

Line -4: Aftab et al should be Hussain et al.

Section 3.2 heading or in contents “optimisation should be optimization”

This paper is a solid contribution to the field of nonlinear analysis, particularly in the context of fixed point problems.

Conclusion: A minor revision is required
